# The First Report of Genetic Polymorphisms of the Equine *SPRN* Gene in Outbred Horses, Jeju and Halla Horses

**DOI:** 10.3390/ani11092574

**Published:** 2021-09-01

**Authors:** Sae-Young Won, Yong-Chan Kim, Kyoungtag Do, Byung-Hoon Jeong

**Affiliations:** 1 Korea Zoonosis Research Institute, Jeonbuk National University, Iksan 54531, Jeonbuk, Korea; gkfh32@jbnu.ac.kr (S.-Y.W.); kych@jbnu.ac.kr (Y.-C.K.); 2 Department of Bioactive Material Sciences and Institute for Molecular Biology and Genetics, Jeonbuk National University, Jeonju 54896, Jeonbuk, Korea; 3 Laboratory of Equine Science, Department of Animal Biotechnology, Faculty of Biotechnology, Jeju National University, Jeju 63243, Korea; challengekt@gmail.com

**Keywords:** prion, scrapie, BSE, CJD, SNP, prion gene family, *SPRN*, *PRNP*, *PRND*, Korean native horse, resistance

## Abstract

**Simple Summary:**

Prion disease is a fatal neurodegenerative disease caused by the accumulation of pathogenic prion protein (PrP^Sc^) in various mammalian hosts. However, to date, prion disease has not been reported in horses. Since the Sho protein encoded by the shadow of the prion protein gene (*SPRN*) plays an essential role in the progression of prion diseases, we investigated the genetic characteristics of the equine *SPRN* gene in horses. We found four single nucleotide polymorphisms (SNPs) of the equine *SPRN* gene and significant different distributions among three horse breeds including Jeju, Halla and Thoroughbred horses. Although the polymorphisms affect the property of mRNA of the equine *SPRN* gene, it did not affect the sequence and structure of Sho protein. Since several non-synonymous SNPs of the *SPRN* gene have been reported in prion diseases-susceptible animals, the absence of non-synonymous SNP of the equine *SPRN* gene in the horses is noticeable.

**Abstract:**

Prion disease is a fatal infectious disease caused by the accumulation of pathogenic prion protein (PrP^Sc^) in several mammals. However, to date, prion disease has not been reported in horses. The Sho protein encoded by the shadow of the prion protein gene (SPRN) plays an essential role in the pathomechanism of prion diseases. To date, the only genetic study of the equine SPRN gene has been reported in the inbred horse, Thoroughbred horse. We first discovered four SPRN single nucleotide polymorphisms (SNPs) in 141 Jeju and 88 Halla horses by direct DNA sequencing. In addition, we found that the genotype, allele and haplotype frequencies of these SNPs of Jeju horses were significantly different from those of Halla and Thoroughbred horses, this latter breed is also included in this study. Furthermore, we observed that the minimum free energy and mRNA secondary structure were significantly different according to haplotypes of equine SPRN polymorphisms by the RNAsnp program. Finally, we compared the SNPs in the coding sequence (CDS) of the SPRN gene between horses and prion disease-susceptible species. Notably, prion disease-susceptible animals had polymorphisms that cause amino acid changes in the open reading frame (ORF) of the SPRN gene, while these polymorphisms were not found in horses.

## 1. Introduction

Prion diseases are chronic and incurable neurodegenerative disorders, and therapeutic substances for these diseases have not been reported to date [1,2]. In prion disease, endogenous prion protein (PrP^C^) is converted to abnormal prion protein (PrP^Sc^), causing spongiform vacuolation and gliosis in the brain [3,4]. To date, the exact cause of conversion of PrP^C^ to PrP^Sc^ has not been reported. However, recently, it has been presumed that *PRNP* genetic mutations, posttranslational modifications, and several genetic factors play a pivotal role in the conversion process of PrP^C^ to PrP^Sc^ [5,6,7,8,9].

Previous studies have reported that among several genetic factors, the prion gene family influences the progression of prion diseases. The prion gene family comprises four members: the prion protein gene (*PRNP)*, the prion-like protein gene (*PRND*), the shadow of the prion protein gene (*SPRN*) and the prion-related protein gene (*PRNT*) [10,11,12,13,14,15]. Among these genes, the Sho protein is encoded by the *SPRN* gene and is structurally similar to the PrP protein. The Sho protein has an N-terminal domain, hydrophobic domain and C-terminal glycosylphosphatidylinositol (GPI) anchor and is a highly conserved protein from fish to mammals [16,17]. In addition, a previous study reported that elevated Sho protein accelerates the progression of prion disease [18]. Furthermore, genetic variations of the *SPRN* gene influence susceptibility to prion disease. In variant Creutzfeldt-Jakob disease (vCJD), the insertion G allele at codon 46 of the human *SPRN* gene induces a frameshift of this gene and it shows a significantly different distribution between the healthy controls and vCJD patients [19]. In goats, a significant association was found between susceptibility to scrapie and the 602_606insCTCCC polymorphism in the 3′ untranslated region (UTR) of the *SPRN* gene [20]. In cattle, insertion/deletion (AAAG) polymorphism was observed in the hydrophobic region of the bovine *SPRN* gene in atypical bovine spongiform encephalopathy (BSE)-affected cattle [21]. These studies indicate that these polymorphisms are related to the expression level that can affect the vulnerability to prion disease in prion disease-susceptible animals.

A previous study has been reported a total of four polymorphisms of the *SPRN* gene in a prion disease-resistant animal, the horse [22]. Notably, the c.87G > C single nucleotide polymorphism (SNP) located in the open reading frame (ORF) was a synonymous SNP and was predicted to have no significant effect on the structural and functional properties of equine Sho protein. However, the investigation of *SPRN* polymorphisms in horses has been performed in only Thoroughbred horses. Since these Thoroughbred horses have been subjected to selective pressure through inbreeding, polymorphism studies in these horses may be limited for representing all horses [23]. Therefore, we investigated polymorphisms of the equine *SPRN* gene in outbred horses, including Jeju and Halla horses.

In this study, we investigated equine *SPRN* polymorphisms using amplicon sequencing in outbred horses, Jeju and Halla horses and compared the genotype and allele frequencies of equine *SPRN* polymorphisms among Jeju, Halla and Thoroughbred horses. We measured the linkage disequilibrium (LD) value of the *SPRN* polymorphisms to estimate indirect statistical epistatic associations among the polymorphisms. Furthermore, we investigated mRNA secondary structure of the equine *SPRN* gene according to the polymorphisms. Lastly, we compared the number of *SPRN* polymorphisms in prion disease-resistant species (horses) and prion disease-susceptible species (humans, cattle, goats and sheep).

## 2. Materials and Methods

### 2.1. Ethical Statement

We collected hair samples from 141 Jeju, 88 Halla and 194 Thoroughbred horses. All experiments were accredited by the Institute of Animal Care and Use Committee of Jeonbuk National University (JBNU 2016-65). All experiments were performed following the Korea Experimental Animal Protection Act.

### 2.2. Animals

The Jeju horse is a Korean wild (outbred) horse native to Jeju Island, South Korea. The Halla horse is a hybrid between the Jeju and Thoroughbred breeds and is bred for racing purposes. According to the Livestock Promotion Agency, 5223 Jeju and 5201 Halla horses have been registered thus far. The samples were collected randomly from animal farms in Jeju Island, South Korea (2018–2019). The Thoroughbred horses have been bred for racing purposes through inbreeding [23]. The samples of Thoroughbred horses were collected randomly from Seoul Race Park in Gwacheon, South Korea (2016–2017) (https://park.kra.co.kr/fromRouter.do?url=https://studbook.kra.co.kr/toRouter.jsp (accessed on 7 November 2016)) [22]. The information on age and sex of animals was not available.

### 2.3. Genetic Analysis

Genomic DNA was extracted from 10 hair bulbs per sample using the HiYieldTM genomic DNA mini kit (Real Biotech Corporation, Taipei, Taiwan) according to the manufacturer’s instructions. In brief, 10 hair bulbs were homogenized with 180 µL of lysis buffer with 20 µL of proteinase K (20 mg/mL) and incubated at 55 °C for 1 h. Then, the DNA was precipitated with absolute ethanol and absorbed to the silica membrane using centrifugation at 13,000 rpm, room temperature for 1 min. The membrane was washed twice with 80% ethanol and eluted with distilled nuclease-free water. The equine *SPRN* gene (Gene ID: 111772531) in horses was amplified using polymerase chain reaction (PCR) with gene-specific forward (AATGCTAAGCTTCTGTCCCCG) and reverse (CTGGTCTTGGCACCTCTCTT) primers. A total of 25 µL of PCR reagent composition was followed by the manufacturer’s protocols. The PCR conditions were 98 °C for 2 min for denaturation; 34 cycles of 98 °C for 20 s, 60 °C for 40 s, and 72 °C for 1 min 30 s; and then 1 cycle of 72 °C for 5 min to extend the reaction. Subsequently, the PCR product was electrophoresed at 120 V for 40 min on a 1% agarose gel with ethidium bromide and visualized in ultraviolet light. The amplicon size was 970 bp. The purification of PCR products was performed using a FavorPrep GEL/PCR purification Mini Kit (FAVORGEN, Pingtung city, Taiwan). Purified PCR products were directly sequenced using an ABI 3730 sequencer (ABI, Foster City, CA, USA); sequencing results were analyzed by Finch TV software (Geospiza Inc., Seattle, WA, USA). The genetic polymorphisms were named based on coding region of the equine *SPRN* gene (Gene ID: 111772531).

### 2.4. mRNA Structural Analysis

‘Mode 1′ of RNAsnp (http://rth.dk/resources/rnasnp/ (accessed on 7 April 2019)) was used to evaluate the effects of the equine *SPRN* polymorphisms on the mRNA structure. RNAsnp estimated the effect of SNPs on the secondary structure of mRNA according to the RNA folding algorithms. ‘Mode 1′ was used to predict the effect of SNPs on short mRNA sequences (<1000 nt). The base pair probabilities by global folding method RNAfold were calculated between wild-type and query RNA sequences. The structural difference between wild-type and query RNA sequences were measured using Euclidean distance or Pearson correlation coefficient for all sequence intervals.

### 2.5. Literature Search

A literature search was carried out to identify studies reporting *SPRN* polymorphisms in humans, cattle, goats, sheep and horses from PubMed. The search terms used were “prion”, “SNP” and “polymorphisms” combined with “human” or “cattle” or “goat” or “sheep” or “horse”. Irrelevant studies were excluded after the initial screening of titles and abstracts. Eligible studies met the following inclusion criteria: (1) Studies regarding the genetic polymorphisms of the *SPRN* gene; (2) full texts; and (3) English language. The exclusion criteria were as follows: (1) Case reports and (2) insufficient genotype data.

### 2.6. Statistical Analysis

Since genotype, allele and haplotype frequencies of the equine *SPRN* polymorphisms were categorical data, the frequencies were compared among three horse breeds, including Thoroughbred, Jeju and Halla horses, by non-parametric tests, chi-square and Fisher’s exact tests using SAS 9.4 software (SAS Institute Inc., Cary, NC, USA). To estimate genetic linkage among *SPRN* polymorphisms, LD analysis was performed using coefficient r^2^ values using Haploview version 4.2 (Broad Institute, Cambridge, MA, USA).

## 3. Results

### 3.1. Comparative Analysis of Genotype and Allele Frequencies of the SPRN Gene among Three Horse Species

**Figure 1:**
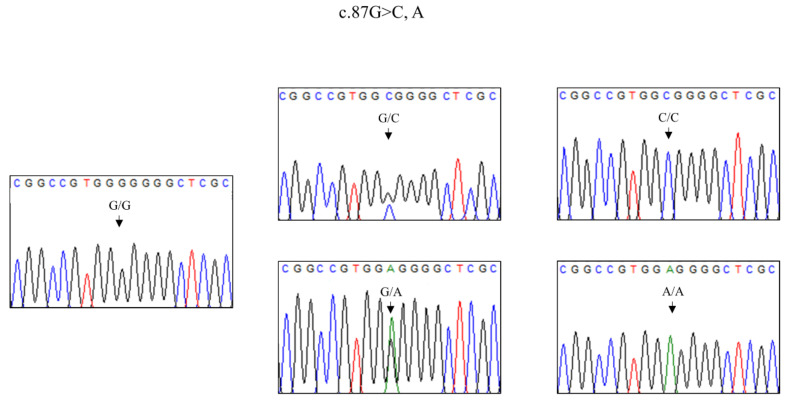
Novel polymorphism of the shadow of the prion protein gene (*SPRN*) identified in Jeju and Halla horses.The electropherogram represents a novel polymorphism, c.87G > A, found in this study. The black arrows indicate the position of SNP. The four DNA nucleotides are represented by different colors: cytosine (blue), thymine (red), guanine (black), and adenine (green).

**Figure 2:**
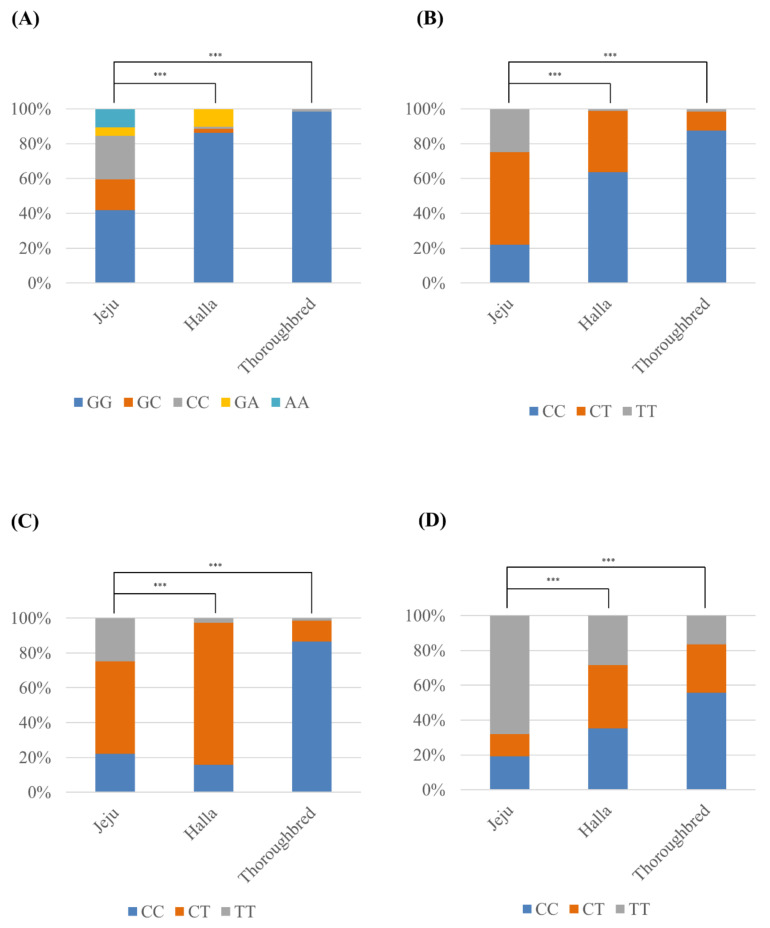
Comparisons of genotype frequency of 4 SNPs identified in 3 horse breeds. The genotype frequencies of c.87G > C, A (**A**), c.502C > T (**B**), c.696C > T (**C**) and c.728C > T (**D**) were compared among the Jeju, Halla and Thoroughbred horses. The difference in genotype frequency was calculated by the chi-square test. Statistically significant differences are indicated. ***: *p*-value < 0.001.

In a previous study, we found a total of 4 *SPRN* SNPs, including c.87G > C, c.502C > T, c.696C > T and c.728C > T, in inbred Thoroughbred horses [22]. In the present study, we first reported 4 *SPRN* SNPs in outbred Jeju and Halla horses, including c.87G > C, A, c.502C > T, c.696C > T and c.728C > T. Notably, c.87G > A (G29G) was a novel synonymous SNP found in Jeju and Halla horses (Figure 1). Detailed information on the genotype and allele frequencies of the *SPRN* SNPs of Jeju and Halla horses is described in Figure 2 and Table 1, respectively.

Next, we compared the genotype and allele frequencies of the equine *SPRN* polymorphisms among Jeju, Halla and Thoroughbred horses. In c.87G > C, A, c.502C > T, c.696C > T and c.728C > T, the genotype and allele frequencies of these SNPs of Jeju horses were statistically different from those of Halla (*p* < 0.001) and Thoroughbred horses (*p* < 0.001) (Figure 2, Table 1).

### 3.2. Haplotype Analysis of the SPRN SNPs in Three Horses

We performed haplotype analysis of the *SPRN* SNPs in Jeju and Halla horses. Eight and 6 major haplotypes were found in Jeju and Halla horses, respectively. The CTTT (30.1%) and GCCC (43.2%) haplotypes showed the highest frequency in Jeju and Halla horses, respectively. Significant differences in CTTT and GCCC haplotypes were detected between Jeju and Halla horses (*p* < 0.0001) (Table 2). In addition, the distributions of the ATTT and CTTC haplotypes showed a significant difference between the Jeju and Halla horses (*p* < 0.01). We also compared the haplotype frequencies between Jeju and Thoroughbred horses. Notably, except for CTTT and GTTC haplotypes, 6 haplotypes including GCCT, GCCC, GTTT, ACCT, ATTT and CTTC haplotypes showed significant differences between the Jeju and Thoroughbred horses (*p* < 0.0001).

### 3.3. LD Analysis of SPRN Polymorphisms in Jeju and Halla Horses

We performed LD analysis among 4 *SPRN* SNPs in Jeju and Halla horses. The c.87G > C SNP showed strong LD with c.502C > T and c.696C > T SNPs in Jeju horses (r^2^ value > 0.3). However, the c.87G > C SNP showed weak LD with c.502C > T and c.696C > T SNPs in Halla horses (r^2^ value < 0.3). In addition, strong LD was found between c.502C > T and c.696C > T in Jeju and Halla horses (r^2^ value > 0.3). Detailed information on the LD analysis is described in Table 3.

### 3.4. mRNA Secondary Structure Analysis of the Equine SPRN Gene According to the Polymorphisms

**Figure 3:**
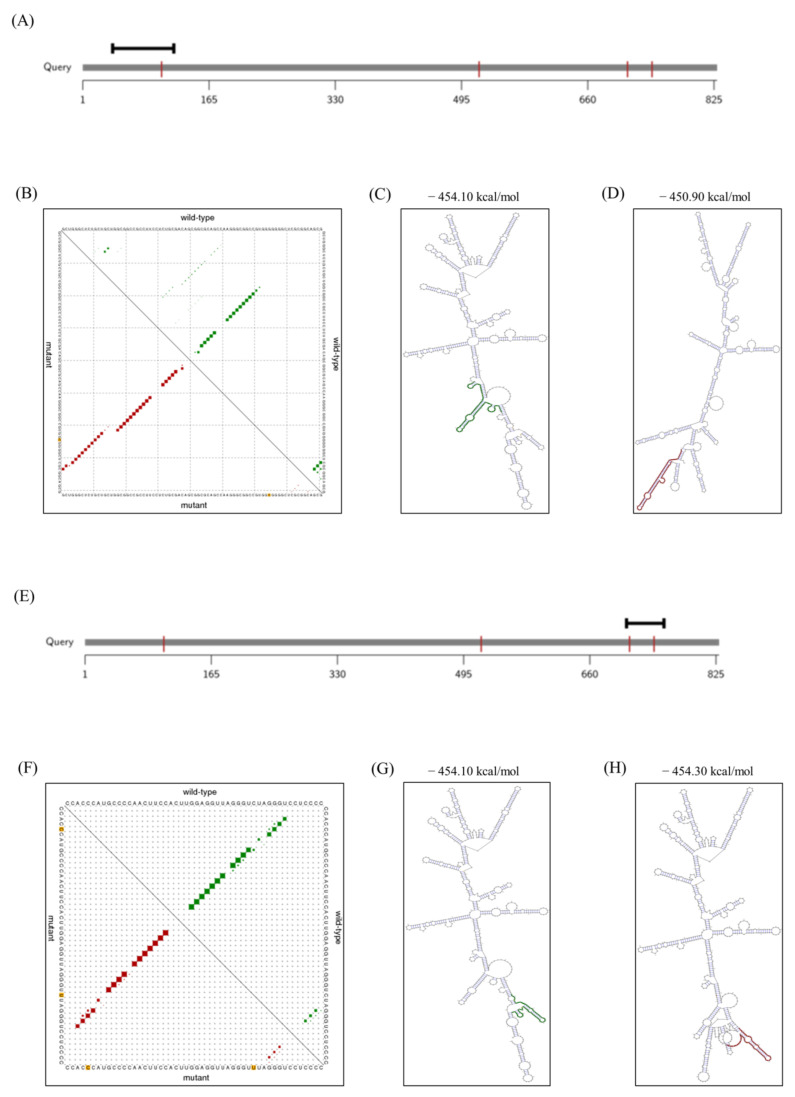
Comparisons of mRNA secondary structure of the equine *SPRN* gene between GCCC and CTTT haplotypes, and between GCCC and GCCT haplotypes. (**A**) The graphical overview shows the location of the predicted local region over the query sequence (GCCC vs. CTTT haplotypes) (**B**) Dot plot represents the base pair probabilities of the mRNA secondary structure. The upper triangle represents the probabilities for the equine *SPRN* gene with GCCC haplotype (green) and the lower triangle represents those for the equine *SPRN* gene with CTTT haplotype (red). (**C**) Minimum free energy and mRNA secondary structure of the equine *SPRN* gene with GCCC haplotype. (**D**) Minimum free energy and mRNA secondary structure of the equine *SPRN* gene with CTTT haplotype. (**E**) The graphical overview shows the location of the predicted local region over the query sequence (GCCC vs. GCCT haplotypes). (**F**) Dot plot represents the base pair probabilities of the mRNA secondary structure. The upper triangle represents the probabilities for the equine *SPRN* gene with GCCC haplotype (green) and the lower triangle represents those for the equine *SPRN* gene with GCCT haplotype (red). (**G**) Minimum free energy and mRNA secondary structure of the equine *SPRN* gene with GCCC haplotype. (**H**) Minimum free energy and mRNA secondary structure of the equine *SPRN* gene with GCCT haplotype.

We investigated the alteration of mRNA secondary structure of the equine *SPRN* gene according to the haplotypes of the polymorphisms using RNAsnp program. In GCCC and CTTT haplotypes of the equine *SPRN* polymorphisms, the graphical overview indicates the difference in the 39–119 region (Figure 3A). The dot plot represents the altered pattern of base pair probabilities between GCCC and CTTT haplotypes (Figure 3B). In addition, the minimum free energy (GCCC: −454.10 kcal/mol; CTTT: −450.90 kcal/mol) and mRNA secondary structure showed the difference between GCCC and CTTT haplotypes (Figure 3C,D). In GCCC and GCCT haplotypes, the graphical overview indicates the difference in the 708–757 region (Figure 3E). The dot plot represents the similar pattern of base pair probabilities between GCCC and GCCT haplotypes (Figure 3F). However, the minimum free energy (GCCC: −454.10 kcal/mol; CTTT: −450.30 kcal/mol) and mRNA secondary structure showed the difference between GCCC and CTTT haplotypes (Figure 3G,H).

### 3.5. The Number of Polymorphisms of the SPRN Gene in Prion Disease-Resistant Animals and Prion Disease-Susceptible Animals

**Figure 4:**
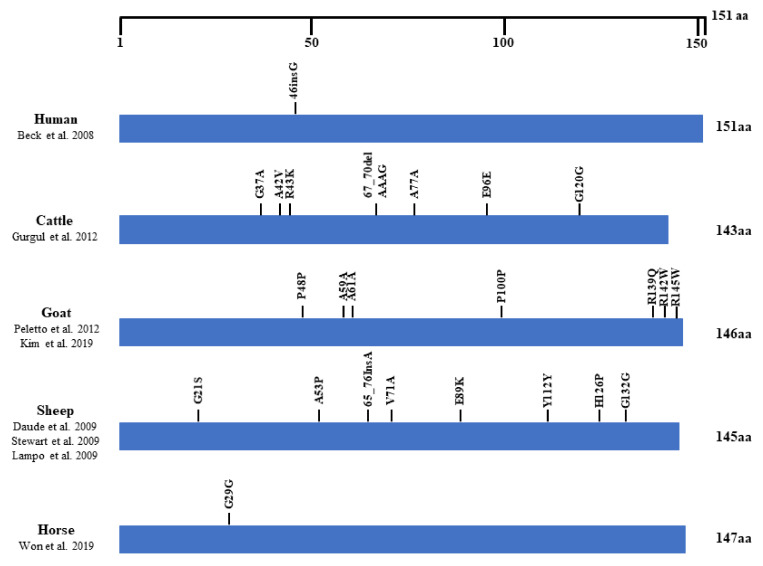
Distribution of polymorphisms in the open reading frame (ORF) of the shadow of the prion protein gene (*SPRN*) in various species. The figure shows the previously reported genetic polymorphisms of the *SPRN* gene in humans, cattle, goats, sheep and horses. The edged horizontal bar indicates the length of the amino acids in the *SPRN* gene [15,19,20,21,22,24,25,26].

We searched the polymorphisms found in the ORF of the *SPRN* gene in prion disease-resistant animals (horse) and prion disease-susceptible animals (human, cattle, goat and sheep) to look for a difference in the number of SNPs between these two groups. Prion disease-susceptible animals had polymorphisms that cause amino acid changes in the OFR of the *SPRN* gene. However, only one synonymous SNP with no change in amino acids was found in prion disease-resistant horses (Figure 4).

## 4. Discussion

Among the prion protein family, Sho proteins, such as PrP, are mainly expressed in the brain and are known to accelerate the progression of prion disease [16,24]. They have been reported to influence embryonic and mammary development [27,28]. It is necessary to investigate the genetic properties of the *SPRN* gene. Since genetic variations of the *SPRN* gene may affect the vulnerability to prion disease via structural changes and the expression level of proteins [19,21,25], we investigated the genetic characteristics of the equine *SPRN* gene. Notably, non-synonymous SNP has not been identified in inbred [22] and outbred horses, and the synonymous SNPs may not affect the structural change of the equine Sho protein. Therefore, the comparison of the role of Sho protein between prion diseases-susceptible animals and horses is needed to confirm the role of equine Sho protein. Previous studies have reported that SNPs of the *PRNP* genes are extraordinarily rare in prion disease-resistant animals, including chickens and horses, compared to prion disease-susceptible animals which denoted highly polymorphic *PRNP* gene [29,30]. In addition, prion disease-resistant animals including dogs and horses showed the weak genetic linkage between the *PRNP* and *PRND* genes compared to prion disease-susceptible animals [13,31]. Therefore, it is necessary to determine whether these genetic features identified in this study are exhibited in other prion disease-resistant animals, including dogs, chickens and rabbits.

We also discovered a novel synonymous SNP, c.87G > A, in the ORF of the *SPRN* gene of the outbred horse breeds, Jeju and Halla horses. Since this SNP has not been found in thoroughbred horses, is is possible that it can be used as a marker to distinguish it from the Thoroughbred horse to the Jeju and Halla horses. A recent study reported that synonymous SNPs affect the transcription, splicing and mRNA stability of genes [32]. Therefore, we investigated the mRNA secondary structure of the equine *SPRN* gene according to the polymorphisms (Figure 3). Although, based on the base pair probabilities, there were no observable significant differences among the three major haplotypes, the minimum free energy and RNA secondary structure showed significant differences according to haplotypes of equine *SPRN* polymorphisms. The minimum free energy and RNA secondary structure can affect translational efficiency [32], and significantly different distributions of synonymous SNPs were identified among horse breeds. Therefore, further analysis of the effects, according to synonymous SNPs of the equine *SPRN* gene on the translation of this gene and the susceptibility to prion diseases in several horse breeds, are needed in the future.

In addition, the Jeju horse showed a significantly different distribution of genotype, allele and haplotype frequencies of the *SPRN* SNPs compared to the Halla horse. Notably, Jeju horses showed greater variability of polymorphisms (Table 1, Figure 2), and it indicates that the conservation of genetic resources is observed in Jeju horses compared to Halla horses. Furthermore, the LDs of the *SPRN* SNPs were different between Jeju and Halla horses. It can be inferred that the *PRNP* gene represents a structural difference in LD blocks between Jeju and Halla horses. Compared to Jeju horse, several distinct characteristics including different genotype, allele and haplotype frequencies, and LD were observed in Halla horse. Since the Halla horse is a crossbred of Jeju and Thoroughbred horses, the genetic characteristics of Halla horse may be influenced by genetic background of these horses. We also found three SNPs in the 3′UTR of the equine *SPRN* gene. The 3′UTR contains the binding site of microRNAs (miRNAs), which play important roles in the regulation of gene expression, and genetic polymorphisms of the 3′UTR are related to the expression level of a gene [33]. Since the expression level of Sho protein is related to the progression of prion diseases and developmental function, functional study of the equine *SPRN* SNPs found in the 3′UTR is needed in further studies.

## 5. Conclusions

In conclusion, we identified four synonymous SNPs of the equine *SPRN* gene and significantly different distributions of genotype, allele and haplotypes among three horse breeds, including Jeju, Halla and Thoroughbred horses. Although the polymorphisms caused alterations of the minimum free energy and secondary structure of mRNA of equine *SPRN* gene, it did not affect the amino sequences and structure of Sho protein. Since several non-synonymous SNPs of the *SPRN* gene were reported in prion diseases-susceptible animals, the absence of non-synonymous SNP of the *SPRN* gene in the horses is noticeable.

## Figures and Tables

**Table 1:** Allele frequencies of shadow of prion protein gene (*SPRN*) SNPs in Jeju, Halla and Thoroughbred horses.

Polymorphisms	Breeds	Total, *n*	Allele Frequency, *n* (%)			*p*-Value	References
c.87G > C			G	C	A		
	Thoroughbred	194	382 (98.5)	6 (1.5)	0 (0)	<0.0001	[22]
	Halla	88	163 (92.6)	4 (2.3)	9 (5.1)	<0.0001	Current study
	Jeju	141	150 (53.2)	95 (33.7)	37 (13.1)	-	Current study
c.502C > T			C	T			
	Thoroughbred	194	361 (93.1)	27 (6.9)		<0.0001	[22]
	Halla	88	143 (81.3)	33 (18.7)		<0.0001	Current study
	Jeju	141	137 (48.6)	145 (51.4)		-	Current study
c.696C > T			C	T			
	Thoroughbred	194	359 (92.5)	29 (7.5)		<0.0001	[22]
	Halla	88	143 (81.3)	33 (18.7)		<0.0001	Current study
	Jeju	141	137 (48.6)	145 (51.4)		-	Current study
c.728C > T			C	T			
	Thoroughbred	194	270 (69.6)	118 (30.4)		<0.0001	[22]
	Halla	88	94 (53.4)	82 (46.6)		<0.0001	Current study
	Jeju	141	72 (25.5)	210 (74.5)		-	Current study

**Table 2:** Haplotype frequency of four shadow of prion protein gene (*SPRN*) SNPs in Jeju, Halla and Thoroughbred horses.

Haplotype	Frequency			*p*-Value ^1^	*p*-Value ^2^
	Jeju (*n* = 282)	Halla (*n* = 176)	Thoroughbred (*n* = 388)		
CTTT	85 (0.301)	9 (0.051)	6 (0.015)	<0.0001	<0.0001
GCCT	60 (0.213)	53 (0.301)	105 (0.271)	0.0997	0.0862
GCCC	38 (0.135)	76 (0.432)	251 (0.647)	<0.0001	<0.0001
GTTT	24 (0.085)	15 (0.085)	5 (0.013)	0.9967	<0.0001
ACCT	23 (0.082)	9 (0.051)	0 (0)	0.2451	<0.0001
ATTT	14 (0.050)	0 (0)	0 (0)	0.0015	<0.0001
CTTC	13 (0.046)	0 (0)	0 (0)	0.0026	<0.0001
GTTC	13 (0.046)	4 (0.023)	14 (0.036)	0.3092	0.5151
Others	12 (0.042)	10 (0.057)	7 (0.018)	-	

*p*-value ^1^: Jeju vs. Halla horses; *p*-value ^2^: Jeju vs. Thoroughbred horses.

**Table 3:** Linkage disequilibrium (LD) among four SNPs of the shadow of prion protein gene (*SPRN*) gene in outbred horses, Jeju and halla horses.

	Jeju				Halla			
r^2^	c.87G > C (Codon 29)	c.502C > T (Codon 168)	c.696C > T (Codon 232)	c.728C > T (Codon 243)	c.87G > C (Codon 29)	c.502C > T (Codon 168)	c.696C > T (Codon 232)	c.728C > T (Codon 243)
c.87G > C (codon 29)	-	-	-	-	-	-	-	-
c.502C > T (codon 168)	0.543	-	-	-	0.098	-	-	-
c.696C > T (codon 232)	0.543	1.0	-	-	0.098	1.0	-	-
c.728C > T (codon 243)	0.072	0.052	0.052	-	0.033	0.01	0.01	-

## Data Availability

Data are available on reasonable request. Requests may be made to bhjeong@jbnu.ac.kr.

## Abbreviations

PrP^C^	Endogenous prion protein
PrP^Sc^	Pathogenic prion protein
*PRNP*	Prion protein gene
*PRND*	Prion-like protein gene
*PRNT*	Prion-related protein gene
*SPRN*	Shadow of prion protein gene
LD	Linkage disequilibrium
GPI	Glycosylphosphatidylinositol
vCJD	Variant Creutzfeldt-Jakob disease
UTR	Untranslated region
BSE	Bovine spongiform encephalopathy
SNPs	Single nucleotide polymorphisms
ORF	Open reading frame
